# Making the call: how does perceived race affect desire to call the police?

**DOI:** 10.1007/s11292-023-09571-z

**Published:** 2023-05-12

**Authors:** Justin L. Sola, Charis E. Kubrin

**Affiliations:** Department of Criminology, Law and Society, University of California, Irvine, USA

**Keywords:** Racial inequality, Racial threat, Calls for service, Political polarization, Police allocation, Preregistered survey experiment, Liberation hypothesis, Crime measurement

## Abstract

**Objectives:**

There is little scholarship about what affects calls for service, even as they originate the vast majority of police interventions in the USA. We test how racial perceptions, ambiguous situational contexts, and participant demographics affect desire to call the police.

**Methods:**

We conduct a nationwide survey experiment with 2,038 participants, varying vignette racial composition (subjects described as black or white) and seriousness of event (less serious, more ambiguous or more serious, less ambiguous) to test two outcomes: 1) desire to call the police and 2) perceived threat.

**Results:**

Perceived race does not directly affect mean desire to call the police or perceived threat. However, political views moderate the effects of race: compared to politically moderate participants, very liberal participants express less desire to call the police while very conservative participants express more desire to call the police in a vignette featuring young Black men.

**Conclusions:**

The political polarization of desire to call the police raises questions about racially differentiated risk of more serious criminal justice system events, including arrest and incarceration, for racial and ethnic minorities.

Calls to 911 for police service (“911 calls” or “calls for service”) originate the vast majority of police interventions and arrests in the USA (Brantingham & Uchida, 2021; Lanfear et al., 2018; Neusteter et al., 2019; Reiss, 1972). The speedy processing of, and constructive response to, such calls underpin effective policing (Blanes i Vidal & Kirchmaier, 2018; Simpson, 2020). At the same time, criminal justice system contact can lead to collateral consequences in the form of cumulative social, economic, and physical harms that disproportionately affect marginalized persons and communities of color (Kurlychek & Johnson, 2019; Martin et al., 2018; Stewart & Uggen, 2019; Sykes & Maroto, 2016; Western, 2018). The decision to call 911 is thus worthy of examination if scholars are concerned with the direct and indirect consequences of police contact on crime, community welfare, and racial inequality (see, for example, Lanfear et al., 2018). At the crux of this dilemma, however, are questions about how calls for service originate, something we know relatively little about.^1^

Historically, research on calls for service has focused on the nature and number of calls, rather than the factors affecting judgment as individuals decide (or not) to call 911. Research reveals that about 50% of calls for service are for non-emergency matters—the majority reflecting noise complaints or requests for information (Antunes & Scott, 1981:168–169)—far outnumbering reports of crime (Antunes & Scott, 1981; Gilsinan, 1989; Meyer, 1974; Neusteter et al., 2019; Sampson, 2004). Beyond cataloging calls, prior studies typically examine the interactional processes of complaint-making and complaint-taking amidst 911 calls (see Antunes & Scott, 1981; Gilsinan, 1989:334; Simpson, 2020; Whalen & Zimmerman, 1990); how community disadvantage, legal institutions, and crime policies affect reliance on, or avoidance of, the police (see Brunson & Wade, 2019; Gau & Brunson, 2009; Louis & Greene, 2020; Schaible & Hughes, 2012; Weitzer & Tuch, 2005); or how violence (Brantingham & Uchida, 2021) or police violence affect calls for service (Desmond et al., 2016).

What remains lesser known is how individuals come to desire police services, and ultimately decide to call for them, particularly in the context of ambiguous situations where a crime may or may not have occurred.^2^ An understanding of desire to call the police is essential given that, as Engel et al. (2012) note, calls for service may be affected by “citizen sensitivity to and intolerance of the conduct of minority group members” (pg. 627). While scholarship has yet to explore such citizen sensitivity, the implications are critical—a sensitivity bias in residents’ desire to call the police may, in turn, bias calls for service, which ultimately affect exposure to the criminal justice system. If biases in desire to call the police result in disparate exposure to the criminal justice system, then assertions that calls for service accurately reflect crime (e.g., Warner & Pierce, 1993:497) are unjustified, and there may be collateral consequences for some groups and communities.

This study investigates desire to call the police. We conduct a nationwide survey experiment with 2,038 participants to examine how racial perceptions, ambiguous situational contexts, and participant demographics affect desire to call the police. In particular, we test the interaction of vignette racial composition (subjects are described as either black or white) and seriousness of event (less serious and more ambiguous, or more serious and less ambiguous) on two outcomes: 1) participants’ desire for police services, and 2) how threatening they perceive the events unfolding in the vignette. By clarifying how personal characteristics and beliefs impact desire to call the police in ambiguous situations, this study helps identify precursors to much criminal justice contact. Through this novel exploration, we invite scholars to critically consider whether calls for service are *prima facie* evidence of crime as “the extent to which the victim and community define a behavior as “criminal” and believe that it is worthy of “outsider” intervention, is perhaps the true extent to which a behavior should be viewed as a crime” (Warner & Pierce, 1993:497).

## Desire to call the police: theoretical foundations and prior research

Scholars suggest that calls for service reflect the most basic form of public cooperation with the police (Brantingham & Uchida, 2021:1; see also Desmond et al., 2016).^3^ Public cooperation with the police, broadly defined, involves actions undertaken to help police secure public safety. Without individual and community cooperation, very few crimes would come to the attention of the police and fewer still would be investigated, cleared, and prosecuted.

At the same time, calling the police is a highly discretionary act on the part of citizens (Gibson et al., 2010:146), resulting in considerable variability in police calls for service across individuals and communities. What explains this variability? Existing research largely focuses on documenting and explaining variability in calls for service across communities, with an eye toward identifying ecological correlates. Among the most robust correlates, studies find that land use matters: blocks with bars (Frisbie et al., 1978; Roncek et al., 1981; Roncek & Pravatiner, 1989) and schools (Perkins et al., 1990; Roncek & Faggiani, 1985) experience higher calls for service (as well as levels of crime) (Kurtz et al., 1998:122). Higher calls for service also occur in communities with greater levels of disorder or incivilities (Hunter, 1978; Kurtz et al., 1998).

Perhaps the most investigated ecological correlate of calls for service is neighborhood socio-economic status. Traditional theoretical perspectives often associate concentrated disadvantage with increased dependence on legal bodies of the state for assistance, intervention, and protection (Schaible & Hughes, 2012:246–247). In this view, agents of the law, such as the police, are seen as substituting for informal social controls in communities lacking adequate institutional strength and support (Schaible & Hughes, 2012:246–247; see also Conklin, 1975; Laub, 1981). Other theoretical perspectives, however, suggest that residents of disadvantaged neighborhoods are unlikely to contact the police for assistance or to report crimes because of limited access to the law (Black, 1976), distrust of the criminal justice system (Schaible & Hughes, 2012:261; see also Anderson, 1999), or due to prior harm from the criminal justice system.

Growing evidence seems to support traditional theoretical perspectives of neighborhood reliance. Using calls-for-service data from a midsize city in the Pacific Northwest, Schaible & Hughes (2012) examine the relationship between neighborhood characteristics and reliance on police for assistance in dealing with serious and minor crimes, as well as physical and social disorder. They find comparatively high levels of police calls for service in disadvantaged neighborhoods, even after controlling for crime. Schaible & Hughes (2012) conclude, “Members of disadvantaged communities have not totally given up on the police and their services” (pg. 262).

St. Louis & Greene (2020) also examine how police services are requested in structurally disadvantaged communities. In particular, they observe the impact of perceptual and structural neighborhood characteristics on crime reporting, feelings of safety, and satisfaction with the police. St. Louis & Greene (2020) find that residents of disadvantaged, high-crime communities request the police more often (consistent with Schaible & Hughes, 2012) yet these same residents *perceive* themselves as unwilling to report crime.

Beyond the above-mentioned research, a handful of studies examine a range of other issues related to police calls for service including how, across multiple cities and for many types of incidents, demand for policing changed during the early months of the COVID-19 pandemic (Ashby, 2020:1056); whether police calls for service increased significantly in the week following a homicide (Brantingham & Uchida, 2021); and, whether police calls for service increased or decreased following a high profile instance of police violence (against an unarmed Black man in Milwaukee, Wisconsin in 2004) (Desmond et al., 2016). While this work is informative, the focus of this study is on what remains less understood: individuals’ desire to call the police in ambiguous contexts that have racial markers.

### Individual desire to call the police

Much less work has been done to explain individual variability in desire to call the police, particularly in ambiguous situations and contexts. Still, several perspectives offer various theoretical expectations. First, a procedural justice argument considers prior experience with the police as critical, emphasizing the perceived quality of treatment one receives during police encounters. Individuals who are stopped by the police may have less trust and confidence in them, as a stop may be coercive and is often an unwanted intervention (Gibson et al., 2010:143). Gibson and colleagues (2010) find that experiencing one or more traffic stops in the past year significantly decreased the likelihood of contacting the police for assistance and of reporting a neighborhood problem, net of other demographic characteristics. Importantly, they find that traffic-stop experiences had similar effects on Whites, Blacks and Hispanics (pg. 139).^4^ As such, those who have been stopped by the police in the past should be less likely to call the police for help in the future.

Second, cultural attenuation theorists posit that individuals within disadvantaged neighborhoods that also experience frequent police coercion develop legal cynicism (Sampson & Bartusch, 1998), leading to the belief that law enforcement and legal institutions are incapable and illegitimate. The desire of residents in such communities to rely on police is thus attenuated (e.g., Wilson, 2012). Residents may view some criminalized conduct (e.g., the sale of drugs) as acceptable, meaning they are disinclined to engage authorities that punish such conduct (Anderson, 1999). These communities do not reject “mainstream” value systems (Gibson et al., 2010:143), but may rely less on the police to address disputes and social problems. In contrast, system avoidance scholars posit that the “increasing integration of institutional databases and monitoring practices” (Brayne, 2014: 387) leads individuals (and communities) to avoid relying on formal institutions, resulting in the same outcome: attenuated reliance on the police.

Beyond those suggested by these theoretical arguments, what other personal characteristics may be associated with desire to call the police in ambiguous situations? Research by Gibson et al. (2010), which considers differences in who is likely to contact the police for services using Police-Public Contact Survey data, offers some idea. Gibson and his colleagues identify significant individual-level correlates of asking for assistance or reporting neighborhood problems. These include age (older citizens were more likely to report problems than younger citizens), gender (men were less likely to report asking for assistance and contacting the police for neighborhood problems), and income (those who make less than $20,000 per year were less likely to report asking for assistance and contacting the police than those who make $20,000-$49,999). Beyond this study, however, we know very little about what characteristics differentiate those who are more or less likely to call the police in ambiguous situations.

### Perceived race and calls for service

Given the focus of the current study, what role does perceived race play when it comes to calls for police service? Wacquant (2009b, 2015) argues that neoliberalism enforces ethno-racial domination via increased police and criminal legal system ensnarement. More broadly, scholars conceptualize neoliberalism as a force that can coordinate disparate fields of action (Bourdieu, 1994) to prioritize the penal “right” hand of the state (Wacquant, 2009a, 2015). This linkage of the criminal legal system to capitalist policy stems from the Marxist criminological tradition (Spitzer, 1975) and racial conflict theorists (Blalock, 1967, 1974). Thus, calls for police service, as an origin point of penal attention, are theorized to rely on residents’ racially-inflected norms of belongingness and citizenry. As such, desire for police services may be inflected by perceived race, such that desire to call the police would increase for penalized minority groups under the auspices of neoliberal responsibilization. In other words, race may determine, in part, how people “See Something, Say Something” because heuristic judgments are subject to stereotypes.

Indeed, social media and news media document that individuals call the police to report people of color participating in public life in ways that do not involve criminal behavior (e.g., while at a barbeque, waiting for a friend in Starbucks, bird watching, or taking a college tour) (Lanfear et al., 2018:1075–1076; see especially Victor, 2018). These incidents often resolve without severe consequences, but not always, as the cases of Tamir Rice and Gregory Hill illustrate (Neusteter et al., 2019:10). These episodes invite scholarship to address a question of public policy concern does perceived race bias residents desire to call the police?

At the same time, recent research has found that direct discrimination reflecting individuals' racial threat-based animus may be weakening (Denver & Pickett, 2022; Sugie et al., 2020). This contrasts with an extensive observational and experimental literature of racial bias in the criminal justice system (Hinton & Cook, 2021; Kirk & Wakefield, 2018; Pickett et al., 2022), generating uncertainty as to whether there is aggregate racial bias in residents’ calls for police service, and under what conditions such bias might occur.

The “liberation” hypothesis posits that situational severity and ambiguity matter when assessing guilt and punishment, giving license (“liberation”) for preferences and biases in cases featuring lower severity and higher ambiguity (Kalven & Zeisel, 1966; Spohn & Cederblom, 1991).^5^ Developed to explain differentials in jury trials, scholars have found that when “juries are ‘liberated’ from a clear decision [they] may use non-statutory and even impermissible factors to guide their decision making…. such as race” (Bjerregaard et al., 2017:1018–9). Recent scholarship uses case disposition records to evaluate the interaction between race and sentencing, and either offers mixed results (Bjerregaard et al., 2017; Hester & Hartman, 2017) or finds that greater leeway does “liberate”—leading to greater racial disparities in sentencing (Chen, 2008).^6^ However, no scholarship has tested the interaction of this potential “liberation” effect on resident calls for service. To locate and test this potential bias, we examine the first step of most police attention: calls for service in ambiguous situations.

Our study is informed by Engel et al. (2012) concern that “animus to minorities” underlies calls for service. Such bias might transform calls for service “from a race-neutral measure of suspect behavior into a measure of citizen sensitivity to and intolerance of the conduct of minority group members. To explore this possibility, it is necessary to undertake individual-level studies of the social psychology of citizens’ making calls for service” (627). Our aim is to investigate how US residents differ in their desire to call the police and in perceived threat, by experimentally varying vignettes’ situational severity, ambiguity, and racial composition.

## Current study

The theoretical perspectives and empirical results discussed above generate three hypotheses, which we test in this study.^7^

- Hypothesis 1: Holding vignette severity constant, we predict that desire to call the police and perceived threat will be greater in vignettes featuring young Black men versus vignettes featuring young White men.
- Hypothesis 2: We predict that the greatest *absolute* difference in desire to call the police and perceived threat will be between the low-severity vignette that features young White men and the high-severity vignette that features young Black men.
- Hypothesis 3: We predict that the greatest *relative* difference in desire to call the police and perceived threat will occur between the low-severity black description vignette and the low severity White description vignette. We expect this because the greater ambiguity of the low-severity vignettes will allow participants’ potential preferences, stereotypes, and biases to play a greater role than in the high-severity vignettes.

Subgroup Heterogeneity: Building on prior work (e.g., Gibson et al., 2010; Spohn & Cederblom, 1991) and to facilitate an exploration of subgroup heterogeneity, we assess how desire to call the police interacts with three personal characteristics: participants’ racial identity, gender identity, and political views. These analyses examine salient aspects of participants’ identity, which may inflect their perceived threat and desire to call the police.

## Data collection and experimental design

### Participant recruitment

We piloted a survey instrument in fall of 2021 with 56 undergraduate students at a public university on the West Coast of the US. The pilot had three purposes. First, it verified that moving between levels of vignette severity (see Table 1) resulted in significantly different levels of participants’ perceived threat and desire to call the police. If this were not the case, we could not test our substantive hypotheses. Second, the pilot verified that the vignettes were not too “extreme” such that participants who viewed the same vignette varied in their perceived threat and desire to call the police. Third, the pilot revealed participants’ time-to-completion, which informed the full study’s compensation strategy. We did not use the pilot to assess how racial descriptions affected participants’ desire to call the police or test other hypotheses.

**Table 1:** Audio vignettes, varying racial description and levels of severity & ambiguity

Vignettes	Lower severity, higher ambiguity	Higher severity, lower ambiguity
White description	• You wake up to the noise of a car alarm going off *1.5 s pause and muffled car alarm starts* • Looking out the window, *0.5 s pause* • you see two young white men *tiny pause* • wearing baseball caps and jeans. *1.5 s pause* • They *seem [mild emphasis]* to be arguing outside of the blaring car.* 1.5 s pause* • After watching for 15 s, *0.5 s pause* • you can’t figure out exactly what is going on • *Car alarm continues for 4 s*	• You wake up to the noise of a car alarm going off *1.5 s pause and muffled car alarm starts* • Looking out the window, *0.5 s pause* • you see two young white men *tiny pause* • wearing baseball caps and jeans. *1.5 s pause* • They *seem [mild emphasis]* to be arguing outside of the blaring car.* 1.5 s pause* • Holding what appears to be a gun, *0.5 s pause* • one shatters the car’s front passenger window • *Muffled car glass shatter, car alarm continues for three seconds*
Black description	• You wake up to the noise of a car alarm going off *1.5 s pause and muffled car alarm starts* • Looking out the window, *0.5 s pause* • you see two young black men *tiny pause* • wearing baseball caps and jeans. *1.5 s pause* • They *seem [mild emphasis]* to be arguing outside of the blaring car.* 1.5 s pause* • After watching for 15 s, *0.5 s pause* • you can’t figure out exactly what is going on • *Car alarm continues for 4 s*	• You wake up to the noise of a car alarm going off *1.5 s pause and muffled car alarm starts* • Looking out the window, *0.5 s pause* • you see two young black men *tiny pause* • wearing baseball caps and jeans. *1.5 s pause* • They *seem [mild emphasis]* to be arguing outside of the blaring car.* 1.5 s pause* • Holding what appears to be a gun, *0.5 s pause* • one shatters the car’s front passenger window • *Muffled car glass shatter, car alarm continues for three seconds*

The audio vignette recordings were designed to minimize threats to external validity. The inclusion of a muffling effect on the car alarm noise, the left–right separation of the audio track, and the muffling effect added to the shattering of glass in the high severity vignette help increase the perceived realism of the audio vignette. Stereo (binaural) mixing of vignettes was identical between conditions:

• 30% pan left on narration

• 50% pan right on muffled car alarm (and muffled car glass shatter in high-severity condition)

Following the pilot study, we recruited participants from January 19^th^–24^th^ 2022 for the full vignette study using Amazon Mechanical Turk (MTurk), a platform for the completion of human intelligence tasks (HITs) in return for compensation. A research deployment platform, CloudResearch, was used to manage the deployment of the survey instrument. Approvals, disapprovals (due to attention check failures or attempted repeats of the survey), compensation, and participant communication were handled via CloudResearch’s user interface.

We restricted the participant pool to US residents who were 18 + years of age, had working audio on an electronic device, and were willing to engage in attention checks during the survey. Beyond age and US residency, two further restrictions were applied. First, an approval rating of 95% or above was required on prior MTurk tasks, which is a standard data quality measure (Peer et al., 2014; Pickett et al., 2018). Second, participants were prohibited from retaking the survey to avoid selection biases and treatment contamination (Barnum & Solomon, 2019). MTurk is well-suited for survey experimental instruments if attention checks and data quality measures are used (Crump et al., 2013; Hunzaker & Mann, 2020; Shank, 2016), and it has become increasingly popular for academic and market research (Berinsky et al., 2012; Bohannon, 2016; Dunbar & Charis 2018).

Power analyses indicated a minimum of ~ 450 participants per condition to discern the effects of a moderate Cohen's d with an interaction, such as whether participant gender interacted with the experimental manipulation of race, with 80% statistical power. Given four vignette conditions, we needed a minimum of 1,800 participants to power the study. We exceeded this threshold with 2,038 total participants in our final sample.

### Audio vignette conditions

An experimental approach permits greater control over extraneous variables, so that only the race of the vignette actors and/or severity of the situation is varied. Notwithstanding obvious limitations of simulated experimental studies related to the reduced gravity, emotionality, and complexity that can result in an experimental setting, this approach offers the best method for controlling extraneous factors that may shape participant desire to call the police. While vignettes, like real interactions, carry ambiguities, judgments must be made, and each dimension of an encounter may affect how respondents interpret the event (Seron et al., 2004:677).

Because we are unaware of any study that uses vignette survey experiments or similar designs to examine desire to call the police, we sought out descriptive literature on calls for service, created vignettes based upon what we learned from those studies, and then piloted our vignette variations. A first step in the design of an experimental survey vignette is devising a template consisting of the characteristics of the social object being studied. A vignette template is composed of dimensions that capture a sequence of events that order the description of the social phenomenon under investigation (Seron et al., 2004:668). Constructing vignettes requires a “practical epistemology” (Whalen & Zimmerman, 1990) to successfully convey a scenario to participants engaging in the survey instrument via a smartphone, personal computer, or tablet device. 911 calls for service require a caller and usually elaborate a reason for the call, for example a situation causing concern. The call-taking operator (re)aligns the situation to match the bureaucratic framework of the call-for-service system and organizational framework of the emergency services resources at hand (Simpson, 2020; Whalen & Zimmerman, 1990).

We created four vignettes that varied 1) the racial description of people involved in the incident (either Black or White) and 2) the severity/ambiguity of the incident (low severity and higher ambiguity or high severity and lower ambiguity). Each vignette lasted 27 s. Over the course of a 911 call several distinct elements coalesce (e.g., Simpson, 2020), and we mirror these elements in our vignettes. These elements are a prospective complainant (the participant), a location, a situation of concern, and a time period for the situation of concern (present). Each vignette starts with the participant waking to the sound of a car alarm. The participant subsequently sees two young men arguing outside of the car in question. In the higher severity, lower ambiguity vignettes, after hearing a car alarm sound, the participant sees what appears to be a gun, and one of the two young men breaks a window on the car. In contrast, in the lower severity, higher ambiguity vignettes, the participant does not see what appears to be a gun, the car window is not broken, and scenario simply ends with the car alarm sounding. Table 1 reviews the four vignettes. The audio files are available upon request.

#### The prospective complainant

Because we sample a wide range of participants, we chose to have a wide external “stance” (Whalen & Zimmerman, 1990:474) for participants in our vignettes. Where possible, we avoid assuming a participant’s relationship with the objects or characters within the vignette. For example, rather than describe the car as the “participant’s car,” we describe “a car.” Similarly, we do not assign the participant any responsibility or social position (Whalen & Zimmerman, 1990:478) that might affect desire to call the police. The characters in the vignette have no specified relationship or direct interaction with the participant, which is important because a relationship, particularly intimate, may decrease desire to call the police (Fleury et al., 1998:337). We assume that participants are capable of calling the police, because 97 + % of U.S. adults have their own mobile phone (Pew Research Center, 2021).

#### Place and time

We use the 2^nd^ person to situate the participant as waking up. The exact time is unspecified, but the area in which the participant “wakes up” has a window. We do not specify the location of the interior, or the time as we wanted participants to imagine their own sleeping schedules and locations, which certainly vary across the sample. We assume that, given a window (generally required in US residences—see Mornu, 2020), the participant can view, and hear, the events described in the vignette. We use the present tense to evoke an ongoing situation rather than either a completed scenario or a prospective event.

#### The situation of concern

Crimes involving cars are common enough that people have written newspaper advice columns asking whether race should affect whether they call the police in light of such crimes (Appiah, 2019). We chose to feature vignettes of a car alarm and argument in the low-severity and higher ambiguity vignette. Proximity to cars is common in the USA, and exposure to car alarms is therefore a frequent and ambiguous phenomenon. For the high-severity and lower ambiguity vignette, we added property damage (breaking of a windshield), potential theft, and the presence of a gun. Theft and crimes involving cars are not uncommon, and US gun ownership is common (e.g., Miller et al., 2022; Sola, 2021).

The situations we chose to include needed to have elements of ambiguity—particularly in the low severity condition—because we wanted participants in the low severity vignette to still be able to articulate a non-minimal (greater than 0) desire to call the police, and participants exposed in the high severity vignette a non-maximal (less than 100) desire to call the police on the [0,100] measurement interval. These characteristics were chosen based on a review of the literature, particularly Antunes & Scott (1981), and based on results from the pilot, which confirmed our predictions about vignette severity.

### Survey instrument

The survey instrument was fielded using Qualtrics. Participants first completed a brief screening questionnaire to verify that they were 18 + years of age, resided in the US, and could hear and classify audio files. Participants who indicated they were not 18 + years of age, did not reside in the US, did not have a US-based IP address, did not successfully complete a ReCaptcha prompt, or could not successfully distinguish whether a brief audio clip was a woman introducing herself, classical music, or rock music, were not allowed to proceed.

Next, participants were randomly assigned to one of the four vignette conditions described above. After listening to the vignette, participants completed a multiple-choice attention check question asking them to summarize what happened in the event, with random ordering in the presentation of responses. Participants who failed the attention check were not allowed to complete or retake the survey. Out of 2,890 times the survey was opened, there were 2,038 successful completions (71%). The rate of successful completions was consistent across vignette conditions: 502 (25% of final sample) in the White low-severity, 519 (25%) in the Black low-severity, 517 (25%) in the White high-severity, and 500 (25%) in the Black high severity-vignette.

#### Outcome measures

Standard practice is to use Likert scales for agreement, satisfaction, and other outcome measures of sentiment. We chose to measure perceived threat and desire to call the police with a horizontal sliding-scale measure (recording on a [0,100] interval). We made this choice for two reasons.

First, we chose a sliding scale to minimize imposed measurement error. Participants click and drag a slider so that their sentiments (when neither maximal or minimal) are not unnecessarily quantized as “2,” “3,” or “4” on a 5-step Likert scale (for example). There is no reason to expect that desire to call the police and perceived threat manifests at a few discrete levels between maximal (e.g., “I absolutely desire to call the police”) and minimal (e.g., “I absolutely don’t desire to call the police”). A slider allows us to capture the interval between maximal (internally recorded as 100) and minimal (internally recorded as 0) with less imposed measurement error.

Second, a continuous scale avoids the problem of participants fixating on a “neutral” middle position with odd-count Likert scales (see Bishop, 1987; Guy & Norvell, 1977; Kalton et al., 1980), while also avoiding the converse problem of neutral participants being unable to express a genuine neutral preference with even-count Likert scales (see Nowlis et al., 2002).

Another common practice is to measure participants’ behavioral intentions, rather than desire. In this case, we could have used a binary (yes, no) or trinary (yes, maybe, no) measure of participants’ intent-to-call, perhaps with a time-bound phrasing to increase verisimilitude (e.g., “Would you call the police within the next 5 min?”). There are two reasons we did not measure prospective behavior: concerns related to low validity and greater social desirability bias.

First, behavioral intent measures have limited external validity for the same reason that New Years’ resolutions are unreliable indicators of future behavior. This is because instead of capturing *likelihood*, behavioral intent measures actually measure participants’ momentary account of prospective future behavior. This disjuncture between intent and actual behavior is known: such “talk is cheap” and not a reliable indicator of future behavior in ethnographic (Jerolmack & Khan, 2014), survey, or experimental contexts (Eifler & Petzold, 2019).

Second, social desirability bias may attach more to the expression of intent (a participant indicating that “I will call the police”) than to the expression of desire (a participant *using a slider to indicate their desire to call the police*). This problem is well-known in surveys of participants’ socially undesirable behaviors (e.g., alcoholism–see Latkin et al., 2017). The problem of social desirability bias even affects studies that ask about participants’ past socially desirable behavior (Ansolabehere & Hersh, 2017; Duff et al., 2007; Hanmer et al., 2014). The prospect of social desirability bias is concerning because it amounts to non-random (dependent on outcome) error in the outcome variable.

#### Operationalization of outcome measures

Immediately after vignette exposure and an attention check on the vignette, participants completed the outcome measures: 1) desire to call the police and 2) perceived threat. Desire to call the police is measured by asking participants “In the scenario you just heard, what is your desire to call the police? Use the slider below to show this:”. Accompanying this is a picture showing a smartphone screen with “911” ready to be dialed. The horizontal slider is labeled as “least desire” at the leftmost bound [0] and “most desire” at the rightmost bound [100].

Perceived threat is measured by asking participants “How threatening is the scenario you just heard? Use the slider below to show this:”. The horizontal slider is labeled as 'least threatening' at the leftmost bound [0] and “most threatening” at the rightmost bound [100].

#### Survey questions and sample characteristics

After successfully completing the vignette attention check and two outcome measures, participants completed a series of questions that reflect key measures (see Table 3 in Appendix) potentially correlated with desire to call the police as reflected in the theoretical discussion and literature reviewed earlier. The order of responses for these questions, when possible, was randomized to reduce anchoring biases (e.g., political views, urbanicity, public assistance, marital status, household characteristics). Following this, participants were served a completion notice with a redemption code for input on the MTurk Web site. The final step for participants, including those who failed the attention check, was viewing the contact information and institutional affiliation of the principal investigator. Survey completion time was measured from opening the survey window in Qualtrics to viewing the contact information. Among participants who successfully completed the survey, median time to completion was 5 min and 37 s.

The final sample consisted of 2,038 MTurk participants. The participants were comparable in age, gender, ethnoracial identity, household income, marital patterns, home ownership, urbanicity, and other variables to the US population as a whole (see Table 3 in Appendix for a full set of participant characteristics). Participants were relatively more educated (62% with a 4-year degree or more education) and more liberal (45% liberal or very liberal) than the US population as a whole, which is common among MTurk samples (Barnum & Solomon, 2019; Hunzaker & Mann, 2020; Pickett et al., 2018; Shank, 2016). Because the experimental design features random assignment to treatments, selection bias is not a threat to validity (Wooldridge, 2018).^8^

### Robustness to participant insight and social desirability bias

The final question of the experiment prompted participants to describe their impression of the survey’s research goal, asking “In a sentence or less, what do you think this survey research is about?” This question served two purposes. The first was to help identify participants who evaded limitations on retaking the survey, particularly in conjunction with other indicators (e.g., shared demographic profiles) so that contamination (by seeing multiple vignettes) did not affect internal validity.^9^ The second was to assess participants’ insight into the purpose of the study. This post-survey approach is inspired by Rubin’s (2016) work to assess the risk of demand characteristics and social desirability bias. Reviewing responses reveals that participants had low insight into the study’s purpose (88% reflecting low insight), and that controlling for participants’ level of insight did not significantly affect study results. We include study insight as a covariate in our full models to predict desire to call the police and perceived threat.

## Analytic plan

### Preregistration

Prior to data collection, we preregistered our experimental design, hypotheses, and general analytic strategy with the Open Science Foundation on January 14, 2022 (see Anonymous, 2022 for a link to the preregistration). We tested subgroup heterogeneity via three interactions, as described above: participant race (White and non-White), participant gender (Female and Non-Female) and political views (Very Liberal, Liberal, Moderate, Conservative, Very Conservative).

### Analytical approach

To assess study hypotheses, we employ simple and multiple fractional logit regressions (FLRs) with robust standard errors to generate predicted margins of our outcome variables: desire to call the police and perceived threat. FLRs are generalized linear models that use a logit link function to predict the expected value (also known as conditional mean) of a bounded but continuous (also known as “interval”) outcome, given a vector of explanatory variables (Papke & Wooldridge, 1996; Wedderburn, 1974). FLR coefficients are interpreted as a *predicted effect* on an interval outcome (here a [0,100] interval of desire to call the police or perceived threat), whereas logistic regression coefficients are interpreted as the *effect on probability* for a binomial distribution of outcomes (for example, yes or no responses to “Did you call the police in the last year?”).

**Fig. 1:**
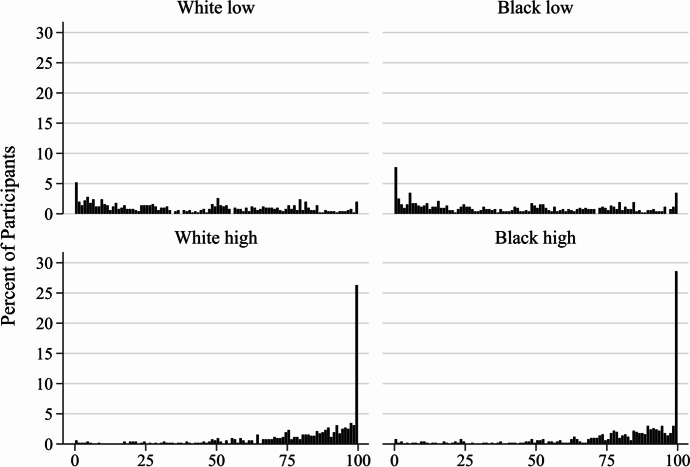
Histograms of desire to call the police by vignette condition (*N* = 2,038)

We use FLRs because the massing at outcome measure boundaries (see Fig. 1) threatens OLS regression assumptions that outcomes are continuous (Ramalho et al., 2011). As quasi-maximum likelihood estimators that are also quasi-parametric, FLRs do not necessitate any particular probability distribution function of the outcome variable (Gourieroux et al., 1984) and are resilient to error non-normality and functional form misspecification (White, 1982:4). Recent interval outcome measures research (e.g., Sola, 2021) confirms prior assessments (e.g., Ramalho et al., 2011) that FLRs outperform OLS regressions.

For all regressions, robust standard errors were used (see Abadie et al., 2023). Multiple regressions used the full set of covariates listed in Table 3 in Appendix (with the exception of sum covariates), along with three additional covariates: logged seconds-to-survey-completion, a measure of participant insight into the research (See *Survey Questions and Sample Characteristics* above), and state fixed effects.

To evaluate how perceived threat mediates the effect of vignette race on participants’ desire to call the police, we use structural equation models to estimate direct, indirect, and total effects (see Zhao et al., 2010). In these models, we use a similar fractional logistic approach with robust standard errors, and control for vignette severity.

To evaluate subgroup heterogeneity via interactions, we use a parallel estimation framework for our regressions and resulting figures (see Bansak, 2021: 73–4; also suggested by Simonsohn, 2019).^10^ Because attitudes towards the police covary with political views, we do not use police legitimacy and police use of force concern covariates when evaluating the interaction of participants’ political views and vignette exposure on desire to call the police and perceived threat. Similarly, when evaluating the interaction of racial identity with the White and non-White indicator variable, we do not use the other ethnoracial covariates.

Data were cleaned and analyses were performed in Stata 17 (Statacorp, 2021) and R (R Core Team, 2022). Additional packages were used to produce tables and figure, including ASDOC (Shah, 2018) in Stata and tidyverse (Wickham et al., 2019) in R. Data and code are available upon request.

## Findings

Recall we suggested that desire to call the police and perceived threat will be greater in high-severity vignettes than in low-severity vignettes. As previously discussed, this aspect of the design was verified in a pilot of a draft survey instrument. High-severity vignettes resulted in significantly greater levels of expected desire to call the police and perceived threat in both simple and multiple regression models (see Table 2), confirming the validity of the vignette severity manipulation, which is necessary to evaluate hypotheses 2 and 3.

**Table 2:** Predicted margins of desire to call the police and perceived threat (*N* = 2038)

*Model*	(1)	(2)	(3)	(4)
*Outcome*	Desire to Call the Police		Perceived Threat	
*Regression type*	Simple	Multiple	Simple	Multiple
White Low-Severity Vignette	41.5	42.5	38.3	38.7
	(1.37)	(1.29)	(1.20)	(1.11)
Black Low-Severity Vignette	42.0	42.0	38.5	38.2
	(1.42)	(1.33)	(1.25)	(1.14)
White High-Severity Vignette	81.9	81.5	72.5	72.5
	(0.97)	(0.96)	(1.02)	(1.01)
Black High-Severity Vignette	81.5	80.8	74.1	73.7
	(1.04)	(1.05)	(0.98)	(0.98)
Covariates				
Demographics	No	Yes	No	Yes
Community views	No	Yes	No	Yes
Police views	No	Yes	No	Yes
State fixed effects	No	Yes	No	Yes

Robust standard errors. **Bolded** confidence intervals indicate that the marginal contrast between the adjoining group is significant (*p* < 0.05). Predicted margins can be interpreted as the average conditional mean of desire to call the police or perceived threat, on a [0,100] interval, after fractional logit regression on covariates (if any)

### *Hypothesis 1*

Recall that hypothesis 1 is that desire to call the police and perceived threat will be greater in vignettes featuring young Black men versus vignettes featuring young White men. As Table 2 reveals, contrary to this hypothesis, Black racial description results in no significant difference in expected desire to call the police or in perceived threat in either simple or multiple regression models. Stated alternatively, we find no expected difference in desire to call the police or perceived threat between vignettes featuring Black young men and vignettes featuring White young men.

### *Hypothesis 2*

Recall that hypothesis 2 is that the greatest *absolute* difference in desire to call the police and perceived threat will be between the low-severity vignette that features young White men and the high-severity vignette that features young Black men. Fractional logit regression analyses show no significant effect of vignette racial description, nor a significant interaction between vignette severity and vignette racial description, on expected desire to call the police or perceived threat, contrary to this hypothesis. Estimates of desire to call the police and perceived threat vary by about one point on a [0,100] scale as vignette racial descriptions vary, a value similar to their standard errors (see Table 2). Therefore, the absolute difference on the basis of racial description posited by this hypothesis is neither substantively nor statistically significant.

### *Hypothesis 3*

Recall that hypothesis 3 is that the greatest *relative* difference in desire to call the police and perceived threat will occur between the low-severity Black description vignette and the low severity White description vignette. We expected this because the greater ambiguity of the low-severity vignettes allows participants’ potential preferences, stereotypes, and biases to play a greater role than in the high-severity vignettes. As discussed, there is no significant difference on the basis of racial description (hypothesis 2), and there is no substantive difference in average conditional mean of desire to call the police or perceived threat on the basis of race (see Table 2). Therefore, the relative difference in desire to call the police and perceived threat on the basis of racial description posited by this hypothesis is neither substantively nor statistically significant.

While the *conditional mean* of desire to call the police does not differ between the White and Black low-severity vignettes, the low-severity Black racial description vignette has the highest standard error of participants’ desire to call the police. We assess this increase in variance in the Subgroup Heterogeneity subsection below.

### Perceived threat mediation

Vignette race has no significant direct, indirect, or total effect on desire to call the police when mediated by perceived threat and controlling for vignette severity. In contrast, vignette severity has highly significant direct, indirect, and total effects (*p*-values < 0.001) on desire to call the police when mediated by perceived threat and controlling for vignette race. These findings show that while perceived threat significantly mediates the effect of vignette severity on desire to call the police, vignette race does not significantly affect participants’ average desire to call the police or perceived threat in either low or high-severity vignettes.

### Subgroup heterogeneity

Overall, findings thus far indicate that desire to call the police and perceived threat are not influenced by vignette racial composition, as originally hypothesized and suggested from extant theory and literature. In particular, our evaluation of hypothesis 1 shows that described race does not significantly affect participants’ average desire to call the police or perceived threat in either low or high-severity vignettes.

At the same time, our evaluation of hypothesis 3 shows that variance in desire to call the police is highest for the Black, low-severity vignette, a finding related to our expectation that the ambiguity of that vignette would increase variance in desire to call the police. Thus, subgroup heterogeneity in desire to call the police may be greater in this vignette condition (see Wang & Ware, 2013 for a discussion of subgroup heterogeneity analysis in experimental contexts). This raises the question: which covariates might drive this variance? Recall that we examine 3 interactions for subgroup heterogeneity: participants’ gender (female versus non-female), racial identity (White versus non-White), and political views (very liberal to very conservative).

### Racial identity

**Fig. 2:**
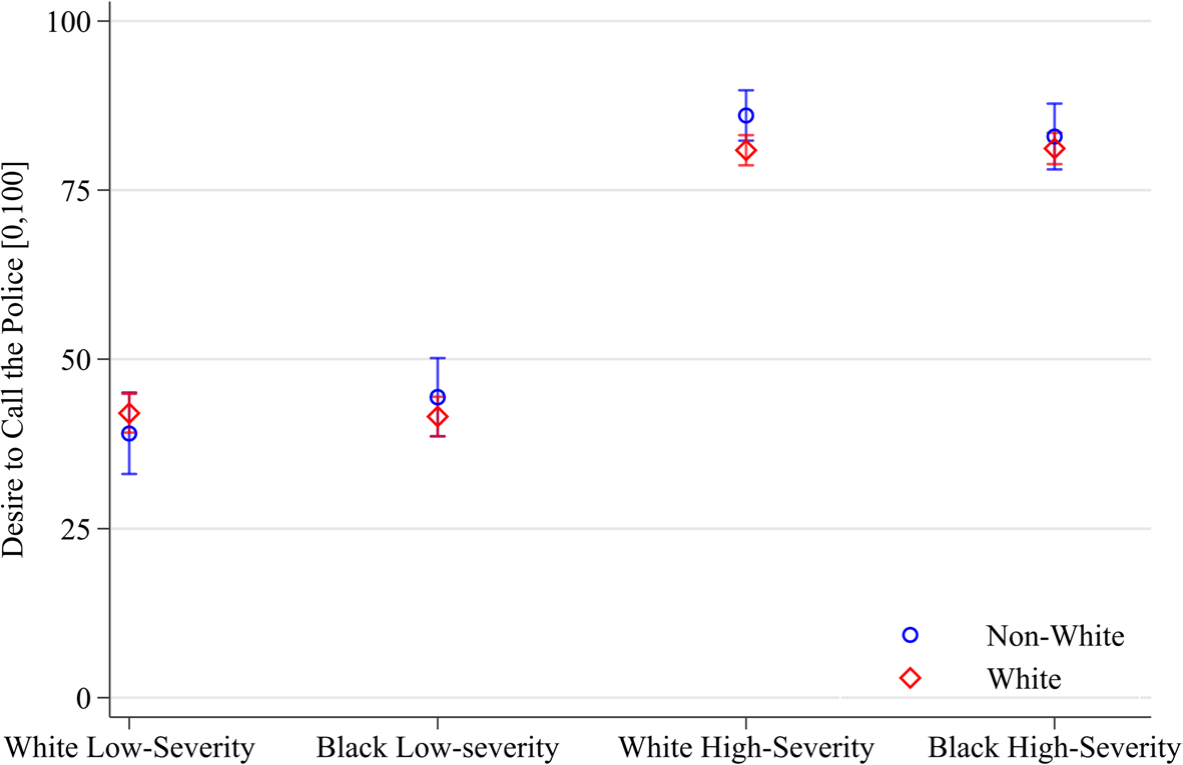
Predicted margins of participant racial identity and vignette interaction on desire to call the police (95% CIs, *N* = 2,038). Note: Robust standard errors. Margins can be interpreted as the average conditional mean of desire to call the police, on a [0,100] interval, for each vignette condition and political view after multiple fractional logit regression with state fixed effects

Participants who identify as White, compared to those who do not, have no significant *direct* differences in their desire to call the police and perceived threat in multiple fractional logit regressions (see Fig. 2). However, predicted margins of desire to call the police and perceived threat differ significantly in the White high-severity vignette condition. White participants express 5.1 points less (95% CI of [− 9.6, − 0.7]) desire to call the police than non-White participants in the White high-severity vignette. However, a test of the equality of coefficients (Holm-Bonferroni corrected) cannot reject the null hypothesis that participants who identify as White are consistent across vignette conditions in their desire to call the police and perceived threat.

### Gender identity

**Fig. 3:**
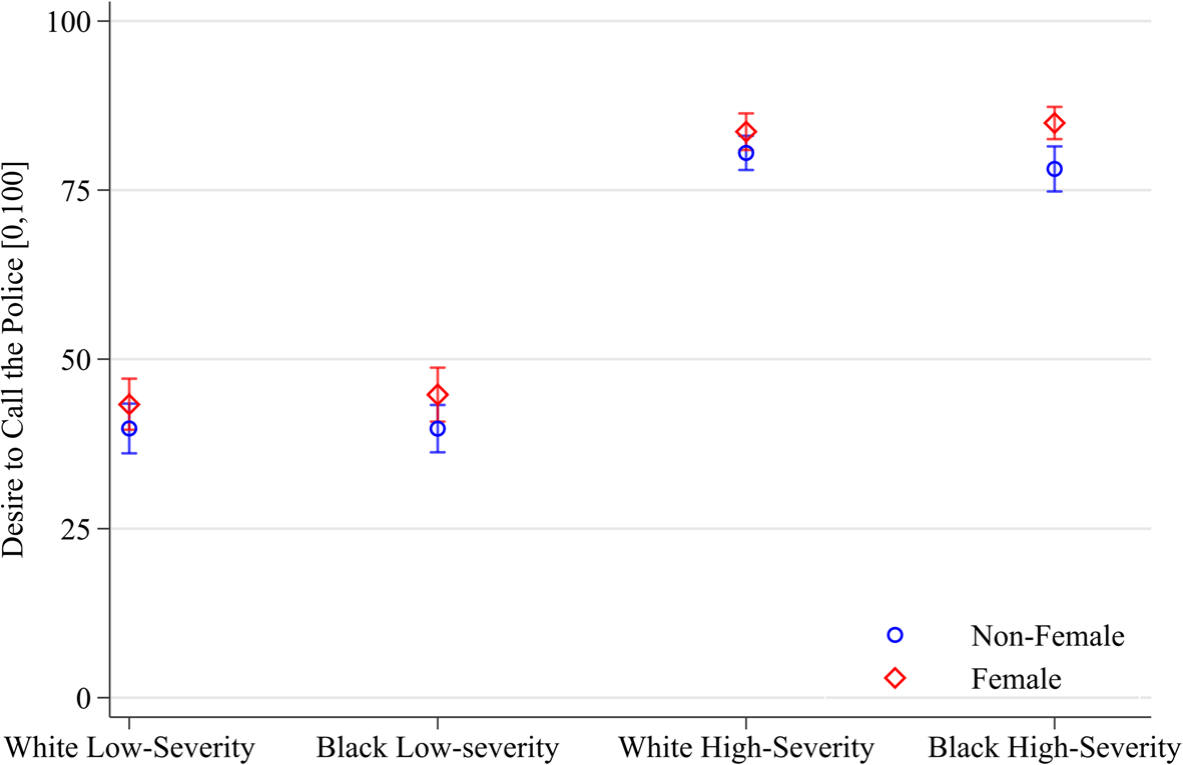
Predicted margins of participant gender identity and vignette interaction on desire to call the police (95% CIs, *N* = 2,038). Note: Robust standard errors. Margins can be interpreted as the average conditional mean of desire to call the police, on a [0,100] interval, for each vignette condition and political view after multiple fractional logit regression with state fixed effects

Participants who identify as female express significantly greater desire to call the police and perceived threat (see Fig. 3). In the multiple logit regressions, participants who identify as female express 4.3 points greater (CI of [+ 1.9, + 6.6]) desire to call the police and 3.3 points greater (CI of [+ 1.1, + 5.5]) perceived threat. A test of the equality of coefficients (Holm-Bonferroni corrected) cannot reject the null hypothesis that participants who identify as female are consistent across vignette conditions in their (greater) expression of desire to call the police and perceived threat.

### Political views

**Fig. 4:**
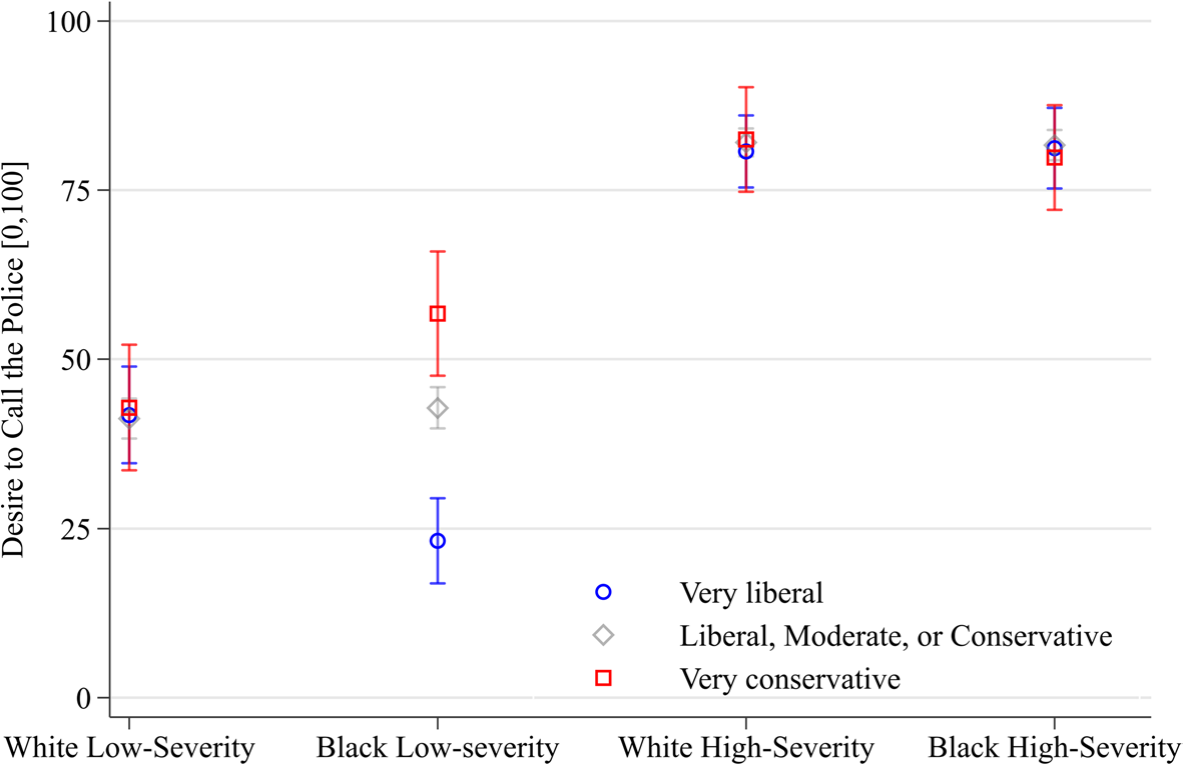
Predicted margins of political views and vignette interaction on desire to call the police (95% CIs, *N* = 2,038). Note: Robust standard errors. Margins can be interpreted as the average conditional mean of desire to call the police, on a [0,100] interval, for each vignette condition and political view after multiple fractional logit regression with state fixed effects

Participants who identify as liberal, moderate, or conservative express desire to call the police (and perceived threat) regardess of vignette racial composition. However, liberal or very conservative diverge significantly from these more moderate participants in the Black low-severity vignette (see Fig. 4). Specifically, we reject the null hypothesis that very liberal and very conservative participants are consistent across vignette conditions in their respective desire to call the police (*p* value of 0.0005, Holm-Bonferroni corrected) and perceived threat (*p*-value of 0.0026, Holm-Bonferroni corrected) across vignette conditions. This finding of political polarization is specific to the Black low-severity vignette.

Very liberal participants express 19.6 points less (95% CI of [− 26.5, − 12.8]) desire to call the police and 16.3 points less (95% CI of [− 22.0, − 10.7]) perceived threat than liberal, moderate, or conservative participants in the Black low-severity vignette. In contrast, very conservative participants express 13.9 points greater (95% CI of [+ 4.2, + 23.6]) desire to call the police and 8.4 points greater (95% CI of [+ 0.1, + 16.8]) perceived threat than liberal, moderate, or conservative participants in the Black low-severity vignette.^11^ See Appendix B for a more complex figure with all five levels of participants’ political views.

## Conclusion

This study investigates desire to call the police. We conducted a nationwide survey experiment with over 2,000 participants to examine how racial perceptions, ambiguous situational contexts, and participant demographics affect desire to call the police. We tested the interaction of vignette racial composition and seriousness of event on participants’ desire for police services, as well as on how threatening they perceive the events unfolding in the vignette. By clarifying how personal characteristics and beliefs impact desire to call the police in ambiguous situations, this study helps identify precursors to much criminal justice contact. This is an important advancement, because it tests bias in the preeminent way that criminal justice system resources are allocated day-to-day: calls for service. Bias in this first step threatens the efficiency and equity of criminal justice system contact following the arrival of police or other law enforcement personnel.

As expected, holding vignette race constant, we find that participants are more likely to perceive threat and desire calling the police in high-severity vignettes than in low-severity vignettes. Consistent with Gibson et al. (2010), we find that women express greater perceived threat and desire to call the police. At the same time, we find that participant race, when dichotomized as White and non-White, does not affect desire to call the police, also consistent with Gibson et al. (2010).

Are individuals more likely to perceive threat and desire to call the police when vignette race is not held constant? Importantly, we find that vignette racial composition does not affect *average* desire of participants to call the police or perceive threat. While contrary to expectations, this finding is reassuring in that there appears to be no significant difference in participants’ average desire to call the police conditional on the racial descriptions in the vignettes.

At the same time, political polarization belies this null result, as desire to call the police and perceived threat diverge in the Black low-severity vignette according to participants’ political views. The Black low-severity vignette is highly salient to the question of bias because of the increased room for interpretation that the low-severity vignette features. As the findings reveal, political polarization increases variance in desire to call the police in this vignette. This finding supports the liberation hypothesis that (lower) situational severity and (higher) situational ambiguity give license liberate) to preferences or biases (Kalven & Zeisel, 1966; Spohn & Cederblom, 1991). Although the liberation hypothesis was developed to explain jury trial outcomes and the court actor decision-making, it may apply to residents’ desire to call the police as well.

How does the higher ambiguity and lower severity of the Black low-severity vignette “liberate” participants? Very liberal participants express less desire to call the police whereas very conservative participants express more desire to call the police, compared to participants who identify as more politically moderate, in the low-severity vignette with Black racial composition. Such polarization is concerning, because it may manifest as racially differentiated risk of exposure to police.

Only one person—out of many observers—is needed to call the police and elicit a response. Therefore, a greater expected variance in desire to call (indicating more people with very high, and very low, desires to call) may result in more calls for service. Therefore, polarization in desire to call the police segments risk of police contact based on chance (who happens to observe?). This is not necessarily problematic; a call for imminent danger or emergency medical services may best be addressed by calling 911.

However, the situation we are referring to—two young Black men arguing beside a blaring car for 15 s—does not appear to constitute imminent threat. The situation is ambiguous, and the description is not criminal. Yet, the results show significant differences in desire to call the police based on participants’ political affiliation. While it is heartening there is no average difference in desire to call the police based on observed race, results also show the opposite—two young Black men arguing outside of a blaring car evoke politically polarized experiences of perceived threat and desire to call the police.

There are two primary limitations to our study. First, we did not observe participant’s real-world calls for police service. This is why we position the study as an extension to, rather than replacement of, the rich literature on calls for police service. Second, this study tests a limited set of audio vignettes and necessarily excludes the potential that different vignettes may have elicited different results. For example, a survey experiment that elicited desire to call the police after presenting vignettes with a different location (e.g., a public park or coffee shop, versus the car alarm featured in this study) may produce different results. Therefore, this study does not offer a conclusive answer to how these alternative vignette specifications would fare. Rather, it serves as an invitation for future research on the nature and extent of biases calls for service. We encourage researchers to use different vignettes and methods to test desire to call the police, testing and refining social theory at every step.

Limitations aside, findings from this study have implications not only for research on calls for service but on related literatures on (racially) inequitable treatment by law enforcement (e.g., Schaible & Hughes, 2012:246). Fear of unfair treatment by the police (see Pickett et al., 2022) can affect how minority groups call the police for help (Avakame et al., 1999; Carr et al., 2007). Inequality in desire for police services may manifest as inequality in exposure to criminal justice system contact. For example, decreased desire for criminal justice system contact may result in “under” policing that harasses local residents but does not address serious crime (St. Louis & Greene, 2020:656; see also Brunson & Wade, 2019).

For an individual, contact with the police increases the risk of more serious criminal justice system events, including arrest, fines and fees (Sykes et al., 2022), incarceration and reentry (Miller, 2021; Western, 2018), and impairments to labor market participation (Sugie et al., 2020; Uggen et al., 2014). This entanglement in the criminal justice system can generate collateral consequences—myriad social, economic, and physical harms that spread to families and communities (Martin et al., 2018; Stewart & Uggen, 2019; Sykes & Maroto, 2016; Western, 2018). Pickett et al. (2022) confirmed prior research (e.g., Gibson et al., 2010:140–6) that there is a significant racial divide in how US residents trust and fear the police. Investigating desire to call the police builds on this research as racially inflected heterogeneity in desire may vary because of racially-disproportionate exposure to negative police-citizen encounters and other adverse traffic stop outcomes (Gibson et al., 2010:142). Our finding of politically polarized desire to call the police should inform future research on police legitimacy.

## Data Availability

Data are available upon request.

## Appendix

Please see Table 3.

**Table 3:** Participant characteristics and covariates (*N* = 2,038)

Variable	Mean	Std. Dev	Min	Max
Desire to call police [0,100]	61.7	33.93	0	100
Perceived threat [0,100]	55.8	30.78	0	100
Age	41.9	13.1	18	85
Gender identity				
Female	47%	0.5	0	1
Male	53%	0.5	0	1
Other identity	0.5%	0.07	0	1
Marital status				
Never married	35%	0.48	0	1
Divorced	8%	0.27	0	1
Separated	1%	0.12	0	1
Widowed	2%	0.12	0	1
Married	48%	0.5	0	1
Domestic partnership	6%	0.24	0	1
Education				
Less than high school	1%	0.08	0	1
High school graduate	10%	0.3	0	1
Some college	18%	0.38	0	1
2-year degree	9%	0.29	0	1
4-year degree	45%	0.5	0	1
Masters or Professional degree	16%	0.36	0	1
Doctorate	1%	0.12	0	1
Employment				
Permanently disabled	1%	0.11	0	1
Retired	6%	0.24	0	1
Student	2%	0.12	0	1
Taking care of home or family	4%	0.18	0	1
Unemployed	5%	0.22	0	1
Temporarily laid off	< 1%	0.04	0	1
Working part time	14%	0.34	0	1
Working full time	69%	0.46	0	1
Household characteristics				
Size of household	2.88	4.27	1	150
Children in household	0.70	1.67	0	40
Household income*****	2.80	2.22	0	10
Political views				
Very liberal	13%	0.33	0	1
Liberal	32%	0.47	0	1
Moderate	25%	0.43	0	1
Variable	**Mean**	**Std. Dev**	**Min**	**Max**
Conservative	22%	0.42	0	1
Very conservative	9%	0.28	0	1
Rent or own				
Rent	36%	0.48	0	1
Own	59%	0.49	0	1
Other housing arrangement	5%	0.22	0	1
Urbanicity				
Rural	18%	0.38	0	1
Suburban	50%	0.5	0	1
Urban	32%	0.47	0	1
Vignette (racial description and severity)				
White, low-severity and high ambiguity	25%	0.43	0	1
Black, low-severity and high ambiguity	25%	0.44	0	1
White, high-severity and low ambiguity	25%	0.44	0	1
Black, high-severity and low ambiguity	25%	0.43	0	1
Racial identity(s) (multiple possible)				
White identity	82%	0.38	0	1
Black identity	19%	0.59	0	2
Native identity	1%	0.11	0	1
Asian identity	8%	0.27	0	1
Pacific identity	< 1%	0.04	0	1
Other racial identity	4%	0.27	0	2
Hispanic identity* (asked separately from racial identity)*	9%	0.29	0	1
Government assistance(s)				
Medical assistance	30%	0.46	0	1
Cash assistance	9%	0.29	0	1
Food assistance	21%	0.41	0	1
Social disability assistance	13%	0.34	0	1
Unemployment assistance	23%	0.42	0	1
Housing assistance	9%	0.28	0	1
Other assistance	4%	0.19	0	1
*Any assistance indicated*	56%	0.5	0	1
Community efficacy variables (0 to 4)				
get along with each other?	3.08	0.79	0	4
are willing to help their neighbor?	2.96	0.93	0	4
share the same values?	2.62	0.95	0	4
can be trusted?	2.79	0.95	0	4
would break up a fight if one broke out in the neighborhood?	2.41	1.06	0	4
*Community efficacy sum [0,20]*	13.86	3.67	0	20
Community crime concern variables (0 to 4)	**Mean**	**Std. Dev**	**Min**	**Max**
Muggings or robberies (in the past year) are a problem	0.77	1.11	0	4
Home burglaries (in the past year) are a problem	0.86	1.09	0	4
Car thefts (in the past year) are a problem	0.97	1.18	0	4
Violent fights (in the past year) are a problem	0.77	1.18	0	4
Sexual assaults (in the past year) are a problem	0.70	1.19	0	4
Shootings or stabbings (in the past year) are a problem	0.78	1.26	0	4
*Community crime concerns sum [0,24]*	4.85	6.37	0	24
Community physical disorder variables (0 to 4)				
Litter, broken glass, or trash on the sidewalks and streets	1.06	1.13	0	4
Vacant or deserted houses, buildings, or storefronts	0.78	1.12	0	4
*Community physical disorder sum [0,8]*	1.84	2.09	0	8
Community social disorder variables (0 to 4)				
People selling or using drugs	1.06	1.27	0	4
Teenagers hanging around on the streets	0.80	1.11	0	4
People being drunk or rowdy in public spaces	0.83	1.19	0	4
*Community social disorder sum [0,12]*	2.69	3.23	0	12
Community precautions variables (0 to 4)				
avoid certain streets or places?	0.93	1.21	0	4
stay in at night?	1.25	1.35	0	4
not travel alone?	0.96	1.23	0	4
carry a weapon?	0.69	1.2	0	4
*Community precautions sum [0,16]*	3.83	4.04	0	16
Crime victimization variables				
Participant was victim of a serious crime (five years)	9%	0.28	0	1
Household member was victim of a serious crime (five years)	6%	0.24	0	1
Police legitimacy variables (0 to 4)******				
Police in my neighborhood are respected	2.93	0.99	0	4
I generally support how police act in my neighborhood	3.01	1.03	0	4
Police in my neighborhood are fair in interactions and decisions	2.93	1.03	0	4
Police are responsive to neighborhood concerns	2.98	1.04	0	4
*Police legitimacy sum [0,16]*	11.85	3.64	0	16
Police concern (over next 5 years) variables (0 to 4)******				
Concern police will stop you	0.88	1.13	0	4
Concern police will search you	0.70	1.13	0	4
Concern police will use insulting language toward you	0.70	1.15	0	4
Concern police will arrest you	0.62	1.13	0	4
Concern police will use physical force against you	0.67	1.16	0	4
Concern police will kill you	0.58	1.15	0	4
*Police concern (over next 5 years) sum [0,24]*	4.14	6.2	0	24
Police contact (over the past 5 years) variables (0 to 3 +)	**Mean**	**Std. Dev**	**Min**	**Max**
Police stops	0.58	0.83	0	3
Police searches	0.25	0.64	0	3
Police insulting language	0.24	0.63	0	3
Police arrests	0.17	0.57	0	3
Police usages of physical force	0.17	0.55	0	3
*Police contact (over past 5 years) sum [0,15]*	1.41	2.63	0	15
Law enforcement affiliation variables				
I work in law enforcement	4%	0.2	0	1
My partner works in law enforcement	3%	0.18	0	1
Close friend(s) works in law enforcement	12%	0.33	0	1
Family member(s) works in law enforcement	11%	0.31	0	1
*Law enforcement affiliation sum [0,4]*	0.31	0.68	0	4

***** Household income measured in $20,000 tranches, with 0 corresponding to “Less than $20,000”, 1 corresponding to “$20,000—$39,999”, 2 corresponding to “$40,000—$59,999”, and 10 corresponding to truncated value of “200,000 or more”

****** These variables are not included in models that interact vignette exposure with participants’ political views, as these variables covary with political views and would overcontrol interactions

## Appendix B – Full predicted margins of political views and vignette interaction on desire to call the police (95% CIs, *N* = 2,038)

Note: Robust standard errors. Margins can be interpreted as the average conditional mean of desire to call the police, on a [0,100] interval, for each vignette condition and political view after multiple fractional logit regression with state fixed effects.

## Footnotes

1 For example, Kurlychek & Johnson (2019) start their annual review of cumulative disadvantage with police discretion, leaving out the important role residents play via their discretionary decisions to call the police.

2 Studies of police contact amidst intimate partner violence (e.g., Fleury et al., 1998) are an exception because they consider process and time-series data of police contact.

3 Calls for service also demonstrate willingness to call the police for help (Gibson et al., 2010), reliance on police services (Schaible and Hughes 2021; St. Louis and Greene 2020), and demand for police services (Ashby 2020).

4 This finding is a bit surprising given the large racial divide in fear of the police (Pickett et al. 2022:302–7) and race differentials in ‘taste’ for punishment (Bobo & Johnson 2004) suggest that an individual’s race differentially affects desire to call the police, something we consider in the current study.

5 Prior to the erection of disciplinary boundaries between sociology and criminology, sociologists of deviance realized that “in an unjust and exploitative society, no matter how "humane" agents of social control are, their actions necessarily result in repression” (Liazos 1972:117).

6 Chen (2008) finds that “wobbler” offenses (which can be discretionarily charged as either a misdemeanor or a felony) correspond to the largest racial sentencing disparities.

7 As discussed below, the hypotheses were preregistered with the Open Science Foundation.

8 A chi-squared test of the balance of covariates across treatment groups shows no significant balance violations.

9 Some participants retook the survey, evading checks by using different MTurk accounts and “incognito” browser windows. Similarities in demographic profiles, web browser configuration, self-described location, and the text-entry question on study purpose were collated programmatically to detect potential retakes. Prior to dropping contaminated results, suspected retakes were manually reviewed.

10 “Interactive effects need interactive controls” because if the “effect of interest is an interaction, any alternative explanation for it must also involve an interaction” (Simonsohn 2019). Specifically, interactions between a randomized treatment and a potential moderator may result in a misestimate if covariates are not also interacted with the treatment (or estimated in parallel—see Bansak 2021).

11 For the Black low-severity vignette, a Chi2 test of equality of perceived threat between 1) very conservative participants and 2) liberal, moderate or conservative results in a *p*-value of 0.0512.
